# Specialized protist communities on mycorrhizal fungal hyphae

**DOI:** 10.1007/s00572-024-01167-3

**Published:** 2024-09-09

**Authors:** Changfeng Zhang, Stefan Geisen, Roeland L. Berendsen, Marcel G. A. van der Heijden

**Affiliations:** 1 Plant-Microbe Interactions, Department of Biology, Faculty of Science, Utrecht University, Padualaan 8, Utrecht, 3584 CH The Netherlands; 2 Plant Soil Interactions, Division Agroecology and Environment, Reckenholzstrasse 191, Agroscope, Zürich, CH- 8046 Switzerland; 3 Physical and Life Sciences Directorate, Lawrence Livermore National Laboratory, Livermore, CA USA; 4 Laboratory of Nematology, Department of Plant Sciences, Wageningen University & Research, Wageningen, The Netherlands; 5 Department of Plant and Microbial Biology, University of Zurich, Zollikerstrasse 107, Zurich, CH-8008 Switzerland

**Keywords:** Arbuscular mycorrhizal fungi, Protists, Cercozoa, Microbiome

## Abstract

Arbuscular mycorrhizal (AM) fungi not only play a crucial role in acquiring nutrients for plants but also serve as a habitat for soil microbes. Recent studies observed that AM fungal hyphae are colonized by specific bacterial communities. However, so far it has not been explored whether fungal hyphae and mycorrhizal networks also harbor specific communities of protists, a key group of microbes in the soil microbiome. Here, we characterized protist communities in soil in a compartment with plant roots and on hyphae collected from hyphal compartments without plant roots. We detected specific protist communities on fungal hyphae. Fourteen protistan amplicon sequences variants (ASVs) were significantly associated with fungal hyphae, half of which belonged to the Cercozoa group. This research, for the first-time detected specific protist ASVs directly associated with abundant AM fungus hyphae, highlighting the complexity of the hyphal food web.

**Supplementary Information:**

The online version contains supplementary material available at 10.1007/s00572-024-01167-3.

## Introduction

Arbuscular mycorrhizal (AM) fungi colonize plant roots and form extensive hyphal networks in the surrounding soil, facilitating the uptake of water and nutrients by the plant (Smith and Read 2010; Martin and van der Heijden 2024). In return, the plants translocate up to 5–20% of their photosynthetic production through the AM fungal hyphae, enriching the surrounding soil with carbon (Jakobsen and Rosendahl 1990; Wang et al. 2022; Hawkins et al. 2023). The interface between AM fungi hyphae and soil is not solely a conduit for chemical exchange but also provides a habitat for a diverse range of soil microbes, including bacteria, archaea and fungi (Zhang et al. 2018, 2024; Emmett et al. 2021; Nuccio et al. 2022). Moreover, recent studies have provided evidence that specific ‘mycorrhiza helper bacteria’ colonize AM fungal hyphae, thereby affecting both the development of mycorrhizal associations and nutrient cycling in the soil (Zhang et al. 2024). Nevertheless, the interactions of AM hyphae with other key groups of soil organisms including protists are still poorly understood.

Protists are abundant in soils, with densities ranging from 10^4^ to 10^8^ per gram (Adl and Coleman 2005). Protists can have various lifestyles, including phototrophy, heterotrophy, mutualism, and parasitism. Phototrophic protists (known as algae) contribute significant amounts of organic carbon to soil (Schmidt et al. 2016), while heterotrophic protists (known as protozoa) consume bacteria and release nitrogen into the soil because of their higher C: N than their prey (Sherr et al. 1983). Furthermore, the nitrogen released by heterotrophic protists into the soil is generally in a form that is readily accessible to plants and leads to enhanced plant growth (Bonkowski 2004; Gao et al. 2019a, b).

De Gruyter et al., (De Gruyter et al. 2022) identified differences in the protist community between pots with and without AM fungi. Moreover, a recent study demonstrated that protists can enhance the utilization of organic nitrogen by AM fungi (Rozmoš et al. 2021). However, it is poorly understood whether protists are associated with AM hyphae in soil, and it is unknown whether AM hyphae harbor specific communities of protists.

Here, we conducted a study using compartmentalized microcosms in a greenhouse study to investigate if AM fungi host specific protist communities. We collected root and soil samples from a plant-colonized compartment and extraradical hyphal samples from another compartment from which plant roots were excluded. We then analyzed the hyphal and soil protist communities using 18 S rRNA gene amplicon sequencing. Our findings provide direct evidence of specialized protist taxa accompanying mycorrhizal fungal hyphae.

## Materials and methods

### Soil collection

The soil used in this study was derived from the Farming System and Tillage experiment (FAST) site (Wittwer et al. 2017, 2021). The FAST site was established in 2009 near Zürich (latitude 47°26′ N, longitude 8°31′ E). In April 2019, soil was collected with the top 2 cm layer of vegetation removed, followed by the excavation of a 20 cm depth of soil from the FAST field. The soil was passed through a 2 mm sieve and stored at 4 ℃ before use.

### Description of microcosms and plant growth conditions

**Fig. 1:**
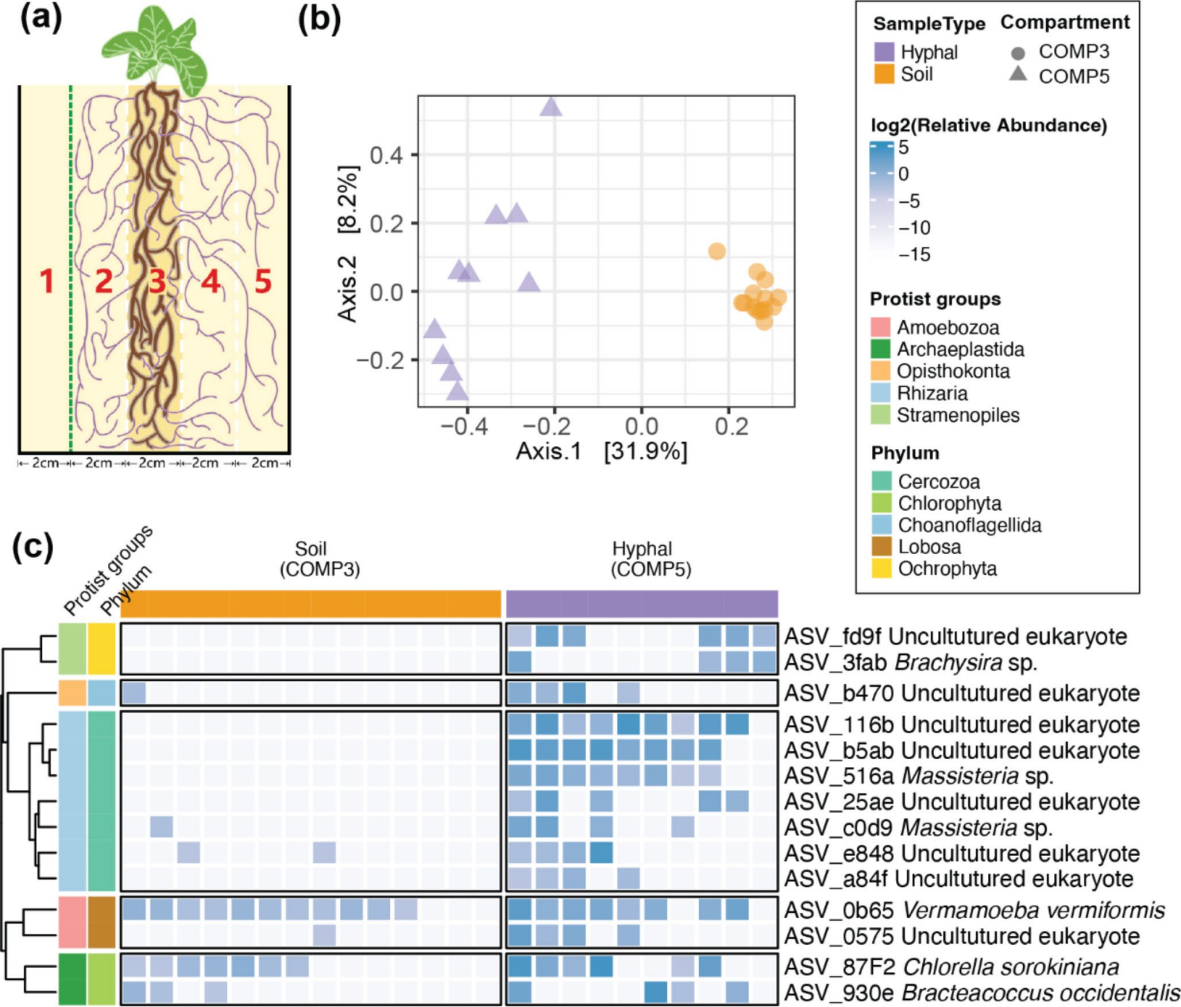
Different protist communities between hyphae and soil. **a** Schematic representation of 5-compartment microcosm layout. COMP3 is filled with 30% unsterilized field soil, whereas COMP1,2,4 and 5 are filled with sterilized soil substrate. Roots are contained in COMP3 by 30 μm meshes (white dashed lines), whereas extraradical fungal hyphae can grow in COMP3, COMP4, and COMP5, but are restricted from COMP1 by a 1 μm filter (green dashed line). **b** PCoA of protist communities in soil (COMP3) and hyphal samples (COMP5). **c** Heatmap of the log2-transformed relative abundance values of protist taxa in soil and hyphal samples. The ASVs presented in the heatmap are significantly (*p* < 0.05) associated with hyphal samples determined by *indicspecies*. The vertical color bars of the heatmap indicate protist groups or phyla. The horizontal color bars of the heatmap indicate sample types and replicates

Microcosms were constructed using frames of 20 × 10 × 19 cm (L×W×H) that were divided in 5 equal compartments (Fig. 1a). The compartments were separated from each other by 30 μm nylon filters that allowed hyphae to pass through but not roots. Compartment 1 (COMP1) was separated by a 1 μm filter that also blocked hyphae. The middle compartment (COMP3) was filled with 1200 g of a mixture of 30% non-autoclaved FAST field soil, 4% autoclaved Oil-Dri (Damolin GmbH, Oberhausen, Germany), and 66% autoclaved sand. This compartment acted as soil inoculum. The outer compartments (COMP1, COMP2, COMP4, and COMP5, respectively) were each filled with 1200 g of the sterilized outer substrate (8% autoclaved FAST field soil, 6% autoclaved Oil-Dri and 86% autoclaved sand). The outer compartments contained a higher fraction of sand to facilitate hyphal collection. COMP2 and COMP4 served as buffer zones to reduce the rhizosphere effect on the neighboring outermost compartments. COMP1 was used as a no-AMF control. COMP5 is the hyphal compartment from which we picked the hyphal samples. We hypothesized that the microbiome in COMP3 was influenced by both roots and hyphae, COMP2 and COMP4 were influenced by root exudates and hyphae, COMP5 was influenced by hyphae alone, and COMP1 was not influenced by either roots or hyphae. The microbiomes of COMP3 and COMP5 were compared to reveal the impact of hyphae on protists while excluding the effect of plant roots on the hyphal microbiome. All autoclaved substrates used in this study were heated to 121℃ for 45 min, twice, with a 24 h interval. Fourteen replicate microcosms were set up.

*Prunella vulgaris* (henceforth Prunella), a plant from the Lamiaceae family, was planted in COMP3. A common plant species in European grasslands, Prunella thrives in the grassland strips of the FAST trial. Chosen as a model species for its size and substantial colonization by AM fungi, Prunella benefits from the association with AM fungi (Van Der Heijden et al. 1998; Streitwolf-Engel et al. 2001; Zhang et al. 2024). Prunella seeds were vapor-phase sterilized by exposure to chlorine gas for 4 h. To this end, chlorine gas was generated by adding 3.2 ml 37% HCl to 100 ml Bleach (Hijman Schoonmaakartikelen BV, Amsterdam, NL). Seed sterilization reduces the variation in the seed microbiome among different seeds. The seeds were sown on half-strength Murashige and Skoog basal agar-solidified medium (Sigma Aldrich, St. Louis, MO, USA). The plates with seeds were subsequently incubated in a climate chamber (Sanyo MLR-352 H; Panasonic, Osaka, Japan) under controlled conditions (light 24 °C 16 h, dark 16 °C 8 h). Seven two-week-old seedlings with roots of approximately ~ 0.5 cm in length that were free of visible contamination were transplanted to each microcosm. Pre-germination of the seeds ensures similar seed size when planting them in the microcosms. The plants in the microcosms were allowed to grow in the greenhouse (Reckenholze, Agroscope, Zürich, CH) with a 16 h photoperiod at 24 °C alternated with 8 h of darkness at 16 °C. Plants were watered with 120 ml H_2_O 2–3 times per week.

### Sampling of fungal hyphae from soil substrate

To sample fungal hyphae, we modified a wet sieving protocol typically used to collect mycorrhizal fungus spores (Pacioni 1992). Briefly, 500 μm, 250 μm, and 36 μm sieves were surface sterilized to minimize contamination by microbes attached to the sieves. The sieves were submersed in 0.5% sodium hypochlorite for 20 min, then submersed in 70% Ethanol for 10 min (Wagg et al. 2014). The sieves were stacked together with the largest filter size on top and the smallest filter size at the bottom. The sieves separate the different sizes of particles making hyphae under microscopy more visible versus non-sieved substrate. So, this sieving method facilitates hyphae extraction from the substrate. Twenty-five grams of soil substrate from COMP5 was placed on the top sieve. The small particles were washed, and soil aggregates were broken with sterilized water. The leftovers on all sieves were washed into Petri dishes. Then, approximately 0.1 ml hyphae were picked from the samples in the Petri dishes using a set of flame-sterilized tweezers under a binocular microscope. The hyphal clumps from different sieves were pooled and analyzed together. We concentrated the hyphae in a single 1.5 ml tube filled with 0.2 ml 30% glycerin per compartment. This was then considered a hyphal sample. The hyphal samples were stored at -80℃ until DNA extraction.

### Soil, root, and hyphal microbiome profiling

The soil and root samples from COMP3 and concentrated hyphae samples from COMP5 were characterized by conducting 18 S amplicon sequencing. DNA extraction from soil, root, and hyphal samples was performed using DNeasy PowerLyzer PowerSoil Kit (Qiagen, Hilden, Germany). The root and soil samples were homogenized in PowerBead solution for 10 min at 30 m/s twice by Tissuelyser II. The hyphal samples were homogenized in PowerBead solution for 2 min at 30 m/s 4 times by Tissuelyser II. The rest of DNA extraction steps of the aforementioned samples followed the manufacturer’s instructions. Extracted DNA was quantified using a Qubit dsDNA BR Assay Kit and Qubit Flex Fluorometer (Thermo Fisher Scientific, Waltham, MA, USA).

DNA was amplified using a two-step PCR protocol. In the initial step, protistan 18 S rRNA genes targeting the V4 region were amplified with the primer set V4_1f (CCAGCASCYGCGGTAATWCC) and TAReukREV3 (ACTTTCGTTCTTGATYRA) (Xiong et al. 2020). The microbial communities were amplified in 24 µl reaction volumes containing 7.5 ng DNA template, 12 µl KAPA HiFi HotStart ReadyMix (F. Hoffmann-La Roche AG, Basel, Switzerland), 0.8 µl 10 µM (protistan) forward and reverse primers and the remaining volume was supplemented by MilliQ-purified water. PCR was conducted by following the cycling conditions of 95 °C for 5 min, followed by 25 cycles of 95 °C for 30 s, 55 °C for 30 s, 72 °C for 45 s, and a final extension at 72 °C for 10 min. The resulting PCR products were purified using AMPure XP beads (Beckman Coulter, High Wycombe, UK) according to the manufacturer’s instructions. The purified PCR products were then used as template DNA in the second PCR. The second PCR was performed in a similar way as above but using primers from the Illumina Nextera Index Kit v2 which contain an error-tolerant 6-mer barcode to allow multiplexed library sequencing. The second-step PCR was conducted by following the cycling conditions of 95 °C for 3 min, followed by 10 cycles of 95 °C for 30 s, 55 °C for 30 s, 72 °C for 30 s, and a final extension at 72 °C for 5 min. The resulting PCR products were cleaned again using AMPure XP beads. The cleaned PCR products were quantified using a Qubit dsDNA BR Assay Kit and Qubit Flex Fluorometer. Equal amounts of PCR product (2 µl 4 nM) were pooled and sequenced on an Illumina MiSeq Sequencer (Illumina, San Diego, USA) using a paired end 300 bp V3 kit at the Utrecht Sequencing Facility (www.useq.nl).

Utilizing ITS amplicon sequencing, we characterized the fungal communities present in soil, root, and hyphal samples. In brief, the fungal ITS2 region was amplified in a 24 µl reaction volume containing 7.5 ng of DNA template, 12 µl of KAPA HiFi HotStart ReadyMix, and 2.5 µl of 2 µM primers (5.8SFun and ITS4Fun) (Taylor et al. 2016; Gao et al. 2019a, b). The remaining volume was supplemented with MilliQ-purified water. The PCR products were then purified using AMPure XP beads. Subsequently, the purified PCR products were amplified using primers from the Illumina Nextera Index Kit v2. Once again, the PCR products were purified with AMPure XP beads. Finally, the purified PCR products were quantified, normalized, pooled, and sequenced. Detailed documentation can be found in the work of Zhang et al. 2024.

### Bioinformatics

Sequence reads were processed in the Qiime2 environment (version 2019.07, https://qiime2.org/) (Bolyen et al. 2019). We used the Demux plugin to assess paired-end sequence quality. The imported primer sequences were removed using Cutadapt (Martin 2011). The paired-end sequences were dereplicated and chimeras were filtered using the Dada2 denoise-paired script (Callahan et al. 2016), which resulted in the identification of amplicon sequence variants (ASVs) and a count table thereof. For ITS sequences, the nonfungal sequences were removed using ITSx (Bengtsson-Palme et al. 2013). 18 S and ITS ASVs were taxonomically annotated employing a pre-trained naive Bayes classifier (Werner et al. 2012) against the PR2 databases (v4.12) (Guillou et al. 2012) and UNITE (v8) database (Kõljalg et al. 2013). From this taxonomic annotation, on average 99.13% of the 18 S ASVs in all root samples were plant ASVs and the root samples were not considered for further analysis. After denoising and filtering of Rhodophyta, Streptophyta, Metazoa, Fungi, and *Embryophyceae* sequences (Xiong et al. 2020; Singer et al. 2021), 347,684 18 S sequences remained from the soil and hyphal samples, and these data was rarefied to a sequence depth of 970 per sample for further analysis (Fig. S1). The fourteen ASVs that were significantly associated with hyphae were blasted against NCBI (https://www.ncbi.nlm.nih.gov/) to confirm their protistan identity.

#### Statistics

All statistical analyses were conducted in R version 4.0.2 (R Core Team 2020). The protist ASV table, the protist taxonomy table and the protist phylogenetic tree generated by Qiime2 were imported to R with Qiime2R (Bisanz 2018). Bray-Curtis distances were calculated and visualized in principal coordinate analysis (PCoA) using the *Phyloseq* package (McMurdie and Holmes 2013). Soil and hyphal protist communities were compared using pairwise permutational analysis of variance (PERMANOVA) performed with the Adonis function in the Vegan package, with 9,999 permutations (Oksanen et al. 2013). *Indicspecies* was used for correlation-based indicator species analysis (Cáceres and Legendre 2009). The visualization of microbial taxonomy and differentially abundant ASVs between sample types used ggplot2 (Wickham 2011) and the Complex Heatmap package (Gu et al. 2016). The sequence abundance was normalized relative to the total number of reads per sample. The comparison of relative abundance between hyphae and soil was determined using the Wilcox test.

## Results and discussion

The protist communities differed between soil and hyphal samples (Fig. 1b). Sample type (hyphal or soil sample) accounted for a significant 30.9% of observed variation between treatments (PERMANOVA, R^2^ = 0.309, F = 9.883, *p* < 0.001). Further investigation was undertaken to identify the specific protist phyla contributing to this difference.

Ochrophyta, often represented by phototrophic algae and belonging to Stramenopiles, were significantly enriched in hyphal samples (RA: 12.5%) compared to soil samples (RA, 3.7%; Fig. S3). Pseudofungi, Apicomplexa, Conosa, Mesomycetozoa, and Chrompodellids exhibited higher abundance in soil samples than in hyphal samples (Fig. S3). It is unresolved what are the specific mechanisms explaining why particular protists are more or less abundant on fungal hyphae. This is linked to the ecological niche of individual protists, their most-important food source (e.g., specific carbon sources or bacteria), the presence or absence of other microbes (competitors, facilitators etc.) and their abiotic niche (including soil texture and water availability).

Subsequently, we investigated the differences of protist ASVs between hyphal and soil samples using *Indicspecies*. A total of 14 ASVs were significantly more abundant on fungal hyphae compared to soil (Fig. 1c, Table S1). Of these 14 hyphal ASVs, 7 were classified as Cercozoa, the protists phylum that generally is most common in soils and mostly bacterivorous (Dumack et al. 2022). Cercozoa were previously also shown to be enriched in rhizospheres (Sapp et al. 2018). Several studies suggest that Cercozoa species have a specific preference for predation on bacteria (Glücksman et al. 2010; Amacker et al. 2022). The bacterial community assembled on fungal hyphae, compared with that of the soil (Zhang et al. 2024), may consequently harbor specific bacterivorous cercozoans that feed on bacteria particularly associated with mycorrhizal fungi. However, it remains uncertain whether these Cercozoa exclusively feed on bacteria attached to hyphae or interact directly or indirectly with fungal hyphae.

Other ASVs that are significantly more abundant on hyphae comprised 2 Ochrophyta ASVs, 2 Chlorophyta ASVs, 2 Choanoflagellida ASVs, and 1 Lobosa ASV (Fig. 1c, Table S1). Only 6 of the 14 hyphal ASVs were identified at the genus level, including *Brachysira*, *Massisteria*, *Vermamoeba*, *Choanoflagellida*, and *Bracteacoccus*. The remaining eight ASVs were classified with an unclear genus designation (Table S1).

The two hyphae enriched Ochrophyta ASVs (ASV_fd9f, ASV_3fab) were identified among all ASVs. Intriguingly, when comparing ASVs only in Ochrophyta between hyphal and soil samples using the Wilcox test, these two ASVs also emerged as the most differentially abundant hyphal ASVs (Fig. S4). ASV_fd9f and AS_3fab exhibit RA of 1.1% and 0.5% in hyphal samples, respectively. Conversely, the RA of these ASVs in soil samples is nearly negligible, approaching zero. This suggests that these two ASVs are the predominant taxa that differentiated Ochrophyta between hyphal and soil samples. To test the preference of these two ASVs for AM fungal hyphae, further studies need to isolate them and experimentally test their function.

**Fig. 2:**
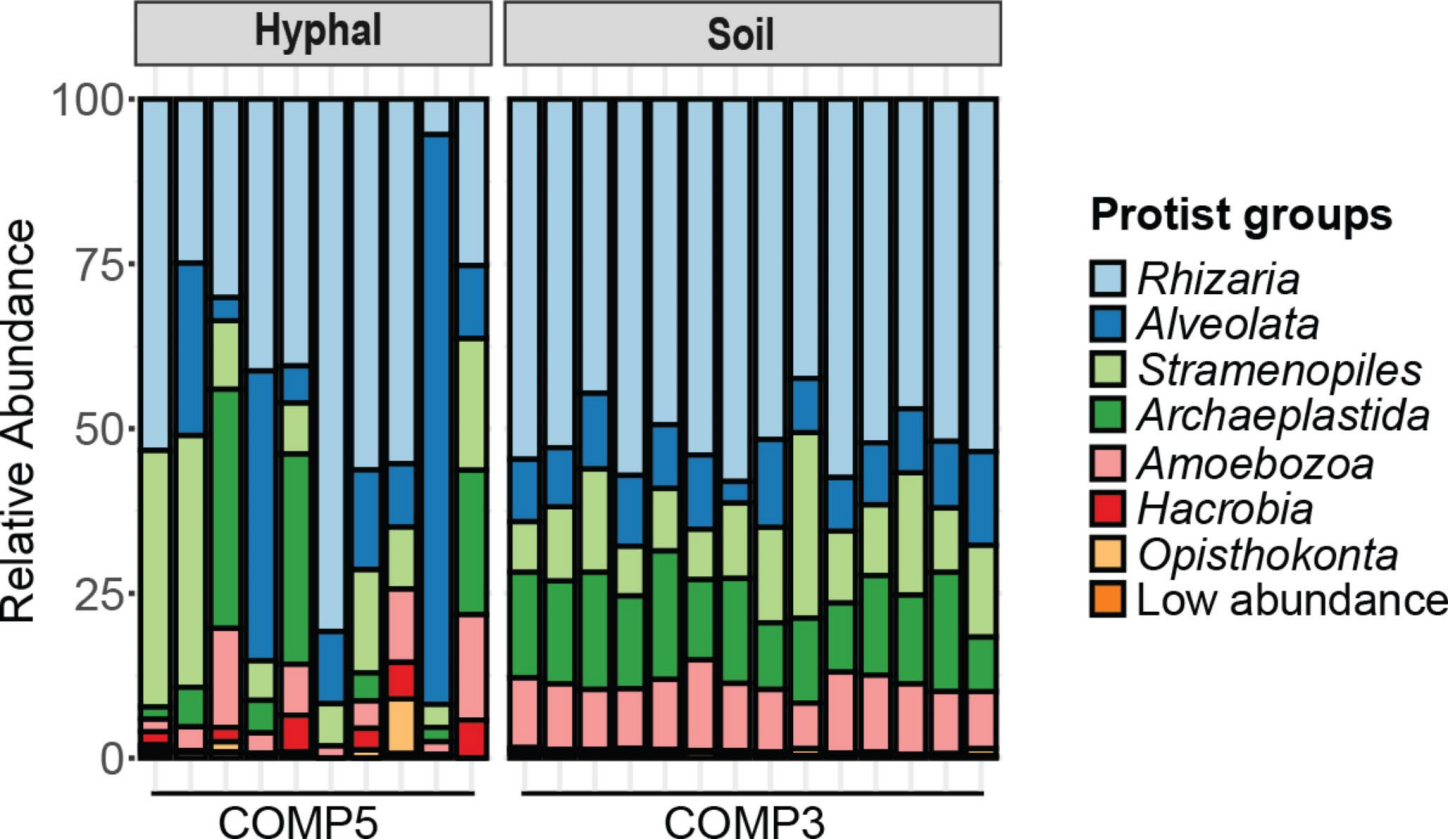
Hyphal and soil samples share common protist groups. Relative abundance of protist groups in soil and hyphal samples. Colors represent different protist groups. The protist groups with relative abundance below 1% were aggregated and categorized as low abundance

Overall, we observed that 7 protist groups together comprised 99.3% of the protist relative abundance (RA) in hyphal samples. These top-seven most-abundant groups were Rhizaria (RA, 41.3%), Alveolata (RA, 21.3%), Stramenopiles (RA, 15.6%), Archaeplastida (RA, 11%), Amoebozoa (RA, 6.6%), Hacrobia (RA, 2.5%), and Opisthokonta (RA, 1.3%; Fig. 2). The abundances of these broad taxonomic groups did not differ significantly between soil and hyphal samples (Fig. S5). From those 7 taxonomic groups, 80 ASVs were detected in both soil and hyphal samples (Fig. S6a). These shared ASVs were abundant in our system with a RA of 64.7% in the hyphal samples and 31.9% in the soil samples (Fig. S6b). This implies that, the majority of the hyphal protist communities are derived from the soil samples.

Using internally transcribed spacer (ITS) amplicon sequencing, we found that the hyphal compartment was colonized by both AM fungi and other non-AM fungi. The relative abundance of *Glomeromycota* fungal phylum (AM fungi) was 51% while the remaining sequences primarily consisted of *Chytridiomycota*, *Ascomycota*, and *Basidiomycota*. Furthermore, within the *Glomeromycota* phylum, two AM fungal species, *Rhizophagus irregularis* (RA: 36%) and *Septoglomus viscosum* (RA: 14%), were most abundant. Detailed data can be found in Zhang et al. 2024. Further studies, thus need to test whether the observed hyphae-specialized protist communities are specific for AM fungi or fungi in general. Note that the soil substrate from the hyphal compartment (COMP5) contained 20% more sand and nearly 80% less soil than the substrate in the plant compartment (COMP3). This was done to facilitate hyphal extraction and to reduce the amount of organic material attached to the fungal hyphae. The varying ratios of soil and sand contribute to differences in substrate texture and nutrient availability. Consequently, disparities in protist communities between hyphae and soil could potentially be because of differences in sand/soil content or differences in the ability of protists to disperse and move through the soil (Zhao et al. 2019). Thus, further studies should verify the observations made here. Moreover, prior to plant growth and mycorrhiza development, the soil substrate in COMP5 was autoclaved to diminish non-AMF fungi, thereby enhancing the likelihood of sampling mycorrhizal fungi originating from COMP3. This may also contribute to the subsequent differences in protist communities between hyphae and soil. This aspect warrants future experiments to validate the conclusions drawn in this study. In addition to collecting hyphae, roots, and soil from different compartments, future studies should also collect hyphae from the compartments with roots to assess whether microbial communities associated with hyphae differ between ‘hyphae-only’ compartments and those associated with root compartments. Alternatively, future studies could collect microbial communities from soil samples taken from the hyphal compartment to avoid any potential bias arising from differences in soil composition.

By extracting protist DNA attached to and surrounding fungal hyphae, our work shows, for the first time, that protists directly colonize fungal hyphae. Furthermore, our work highlights that protist communities developed on fungal hyphae differ from the original protist community in field soil. Further work now needs to test whether protists play a role in the functioning of the plant-AM fungi symbiosis and whether protists may use fungal networks and mycelia as hyphal highways to spread through the soil. These findings highlight the intricate nature of the food web associated with AM hyphae and elucidate a significant connection between AM fungi and their associated microbes.

## Electronic supplementary material

Below is the link to the electronic supplementary material.

Supplementary Material 1

Supplementary Material 2

## Data Availability

The raw sequencing data of 18S were deposited at the European Nucleotide Archive (http://www.ebi.ac.uk/ena) by the study accession PRJEB67506.

## References

[CR1] Adl SM, Coleman DC (2005) Dynamics of soil protozoa using a direct count method. Biol Fertil Soils 42:168–171

[CR2] Amacker N, Gao Z, Hu J, Jousset AL, Kowalchuk GA, Geisen S (2022) Protist feeding patterns and growth rate are related to their predatory impacts on soil bacterial communities. FEMS Microbiol Ecol 98(6):fiac057 35524686 10.1093/femsec/fiac057 PMC9126823

[CR3] Bengtsson-Palme J, Ryberg M, Hartmann M, Branco S, Wang Z, Godhe A, De Wit P, Sánchez‐García M, Ebersberger I, de Sousa F (2013) Improved software detection and extraction of ITS1 and ITS 2 from ribosomal ITS sequences of fungi and other eukaryotes for analysis of environmental sequencing data. Methods Ecol Evol 4(10):914–919

[CR4] Bisanz JE (2018) qiime2R: Importing QIIME2 artifacts and associated data into R sessions. Version 0.99 13

[CR5] Bolyen E, Rideout JR, Dillon MR, Bokulich NA, Abnet CC, Al-Ghalith GA, Alexander H, Alm EJ, Arumugam M, Asnicar F (2019) Reproducible, interactive, scalable and extensible microbiome data science using QIIME 2. Nat Biotechnol 37(8):852–857 31341288 10.1038/s41587-019-0209-9 PMC7015180

[CR6] Bonkowski M (2004) Protozoa and plant growth: the microbial loop in soil revisited. New Phytol 162(3):617–631 33873756 10.1111/j.1469-8137.2004.01066.x

[CR7] Cáceres MD, Legendre P (2009) Associations between species and groups of sites: indices and statistical inference. Ecology 90(12):3566–3574 20120823 10.1890/08-1823.1

[CR8] Callahan BJ, McMurdie PJ, Rosen MJ, Han AW, Johnson AJA, Holmes SP (2016) DADA2: high-resolution sample inference from Illumina amplicon data. Nat Methods 13(7):581–583 27214047 10.1038/nmeth.3869 PMC4927377

[CR26] R Core Team (2020) R: A language and environment for statistical computing. R Foundation for Statistical Computing, Vienna, Austria. https://www.R-project.org/

[CR9] De Gruyter J, Weedon JT, Elst EM, Geisen S, Van der Heijden MG, Verbruggen E (2022) Arbuscular mycorrhizal inoculation and plant response strongly shape bacterial and eukaryotic soil community trajectories. Soil Biol Biochem 165:108524

[CR10] Dumack K, Feng K, Flues S, Sapp M, Schreiter S, Grosch R, Rose LE, Deng Y, Smalla K, Bonkowski M (2022) What drives the assembly of plant-associated protist microbiomes? Investigating the effects of crop species, soil type and bacterial microbiomes. Protist 173(6):125913 36257252 10.1016/j.protis.2022.125913

[CR11] Emmett BD, Lévesque-Tremblay V, Harrison MJ (2021) Conserved and reproducible bacterial communities associate with extraradical hyphae of arbuscular mycorrhizal fungi. ISME J 15(8):2276–2288 33649552 10.1038/s41396-021-00920-2 PMC8319317

[CR12] Gao C, Montoya L, Xu L, Madera M, Hollingsworth J, Purdom E, Hutmacher RB, Dahlberg JA, Coleman-Derr D, Lemaux PG (2019a) Strong succession in arbuscular mycorrhizal fungal communities. ISME J 13(1):214–226 30171254 10.1038/s41396-018-0264-0 PMC6298956

[CR13] Gao Z, Karlsson I, Geisen S, Kowalchuk G, Jousset A (2019b) Protists: puppet masters of the rhizosphere microbiome. Trends Plant Sci 24(2):165–176 30446306 10.1016/j.tplants.2018.10.011

[CR14] Glücksman E, Bell T, Griffiths RI, Bass D (2010) Closely related protist strains have different grazing impacts on natural bacterial communities. Environ Microbiol 12(12):3105–3113 20602629 10.1111/j.1462-2920.2010.02283.x

[CR15] Gu Z, Eils R, Schlesner M (2016) Complex heatmaps reveal patterns and correlations in multidimensional genomic data. Bioinformatics 32(18):2847–2849 27207943 10.1093/bioinformatics/btw313

[CR16] Guillou L, Bachar D, Audic S, Bass D, Berney C, Bittner L, Boutte C, Burgaud G, de Vargas C, Decelle J (2012) The Protist Ribosomal reference database (PR2): a catalog of unicellular eukaryote small sub-unit rRNA sequences with curated taxonomy. Nucleic Acids Res 41(D1):D597–D604 23193267 10.1093/nar/gks1160 PMC3531120

[CR17] Hawkins H-J, Cargill RIM, Van Nuland ME, Hagen SC, Field KJ, Sheldrake M, Soudzilovskaia NA, Kiers ET (2023) Mycorrhizal mycelium as a global carbon pool. Curr Biol 33(11):R560–R573 37279689 10.1016/j.cub.2023.02.027

[CR18] Jakobsen I, Rosendahl L (1990) Carbon flow into soil and external hyphae from roots of mycorrhizal cucumber plants. New Phytol 115(1):77–83

[CR19] Kõljalg U, Nilsson RH, Abarenkov K, Tedersoo L, Taylor AFS, Bahram M, Bates ST, Bruns TD, Bengtsson-Palme J, Callaghan TM et al (2013) Towards a unified paradigm for sequence-based identification of fungi. Mol Ecol 22(21):5271–5277 24112409 10.1111/mec.12481

[CR21] Martin M (2011) Cutadapt removes adapter sequences from high-throughput sequencing reads. EMBnet J 17(1):10–12

[CR20] Martin FM, van der Heijden MGA (2024) The mycorrhizal symbiosis: research frontiers in genomics, ecology, and agricultural application. New Phytol 242(4):1486–1506 38297461 10.1111/nph.19541

[CR22] McMurdie PJ, Holmes S (2013) Phyloseq: an R package for reproducible interactive analysis and graphics of microbiome census data. PLoS ONE 8(4):e61217 23630581 10.1371/journal.pone.0061217 PMC3632530

[CR23] Nuccio EE, Blazewicz SJ, Lafler M, Campbell AN, Kakouridis A, Kimbrel JA, Wollard J, Vyshenska D, Riley R, Tomatsu A (2022) HT-SIP: a semi-automated stable isotope probing pipeline identifies cross-kingdom interactions in the hyphosphere of arbuscular mycorrhizal fungi. Microbiome 10(1):1–20 36434737 10.1186/s40168-022-01391-z PMC9700909

[CR24] Oksanen J, Blanchet FG, Kindt R, Legendre P, Minchin PR, O’hara RB, Simpson GL, Solymos P, Stevens MHH, Wagner H (2013) Community ecology package. R Package Version 2(0):321–326

[CR25] Pacioni G (1992) 16 wet-sieving and decanting techniques for the extraction of spores of vesicular-arbuscular fungi. Methods Microbiol 24:317–322

[CR27] Rozmoš M, Bukovská P, Hršelová H, Kotianová M, Dudáš M, Gančarčíková K, Jansa J (2021) Organic nitrogen utilisation by an arbuscular mycorrhizal fungus is mediated by specific soil bacteria and a protist. ISME Journal(March): 1–10 10.1038/s41396-021-01112-8 PMC8857242 34545172

[CR28] Sapp M, Ploch S, Fiore-Donno AM, Bonkowski M, Rose LE (2018) Protists are an integral part of the Arabidopsis thaliana microbiome. Environ Microbiol 20(1):30–43 28967236 10.1111/1462-2920.13941

[CR29] Schmidt O, Dyckmans J, Schrader S (2016) Photoautotrophic microorganisms as a carbon source for temperate soil invertebrates. Biol Lett 12(1):20150646–20150646 26740559 10.1098/rsbl.2015.0646 PMC4785913

[CR30] Sherr BF, Sherr EB, Berman T (1983) Grazing, growth, and ammonium excretion rates of a heterotrophic microflagellate fed with four species of bacteria. Appl Environ Microbiol 45(4):1196–1201 16346264 10.1128/aem.45.4.1196-1201.1983 PMC242438

[CR31] Singer D, Seppey CV, Lentendu G, Dunthorn M, Bass D, Belbahri L, Blandenier Q, Debroas D, de Groot GA, De Vargas C (2021) Protist taxonomic and functional diversity in soil, freshwater and marine ecosystems. Environ Int 146:106262 33221595 10.1016/j.envint.2020.106262

[CR32] Smith SE, Read DJ (2010) Mycorrhizal symbiosis. Academic

[CR33] Streitwolf-Engel R, Van der Heijden M, Wiemken A, Sanders I (2001) The ecological significance of arbuscular mycorrhizal fungal effects on clonal reproduction in plants. Ecology 82(10):2846–2859

[CR34] Taylor DL, Walters WA, Lennon NJ, Bochicchio J, Krohn A, Caporaso JG, Pennanen T (2016) Accurate estimation of fungal diversity and abundance through improved lineage-specific primers optimized for Illumina amplicon sequencing. Appl Environ Microbiol 82(24):7217–7226 27736792 10.1128/AEM.02576-16 PMC5118932

[CR35] Van Der Heijden MG, Klironomos JN, Ursic M, Moutoglis P, Streitwolf-Engel R, Boller T, Wiemken A, Sanders IR (1998) Mycorrhizal fungal diversity determines plant biodiversity, ecosystem variability and productivity. Nature 396(6706):69–72

[CR36] Wagg C, Bender SF, Widmer F, Van Der Heijden MGA (2014) Soil biodiversity and soil community composition determine ecosystem multifunctionality. Proc Natl Acad Sci USA 111(14):5266–5270 24639507 10.1073/pnas.1320054111 PMC3986181

[CR37] Wang F, Zhang L, Zhou J, Rengel Z, George TS, Feng G (2022) Exploring the secrets of hyphosphere of arbuscular mycorrhizal fungi: processes and ecological functions. Plant Soil 481(1–2):1–22

[CR38] Werner JJ, Koren O, Hugenholtz P, DeSantis TZ, Walters WA, Caporaso JG, Angenent LT, Knight R, Ley RE (2012) Impact of training sets on classification of high-throughput bacterial 16s rRNA gene surveys. ISME J 6(1):94–103 21716311 10.1038/ismej.2011.82 PMC3217155

[CR39] Wickham H (2011) ggplot2. Wiley Interdisciplinary Reviews: Comput Stat 3(2):180–185

[CR40] Wittwer RA, Dorn B, Jossi W, van der Heijden MGA (2017) Cover crops support ecological intensification of arable cropping systems. Sci Rep 7(1):41911 28157197 10.1038/srep41911 PMC5291223

[CR41] Wittwer RA, Franz Bender S, Hartman K, Hydbom S, Lima A, Loaiza RA, Nemecek V, Oehl T, Axel Olsson F, Petchey P O, et al (2021) Organic and conservation agriculture promote ecosystem multifunctionality. Sci Adv 7 10.1126/sciadv.abg6995 PMC8378818 34417179

[CR42] Xiong W, Song Y, Yang K, Gu Y, Wei Z, Kowalchuk GA, Xu Y, Jousset A, Shen Q, Geisen S (2020) Rhizosphere protists are key determinants of plant health. Microbiome 8:1–9 32127034 10.1186/s40168-020-00799-9 PMC7055055

[CR44] Zhang L, Shi N, Fan J, Wang F, George TS, Feng G (2018) Arbuscular mycorrhizal fungi stimulate organic phosphate mobilization associated with changing bacterial community structure under field conditions. Environ Microbiol 20(7):2639–2651 29901256 10.1111/1462-2920.14289

[CR43] Zhang C, van der Heijden MGA, Dodds BK, Nguyen TB, Spooren J, Valzano-Held A, Cosme M, Berendsen RL (2024) A tripartite bacterial-fungal-plant symbiosis in the mycorrhiza-shaped microbiome drives plant growth and mycorrhization. Microbiome 12(1):13 38243337 10.1186/s40168-023-01726-4 PMC10799531

[CR45] Zhao Z-B, He J-Z, Geisen S, Han L-L, Wang J-T, Shen J-P, Wei W-X, Fang Y-T, Li P-P, Zhang L-M (2019) Protist communities are more sensitive to nitrogen fertilization than other microorganisms in diverse agricultural soils. Microbiome 7:1–16 30813951 10.1186/s40168-019-0647-0 PMC6393985

