# Supplementary material for: Specialized protist communities on mycorrhizal fungal hyphae
Source: Mycorrhiza. 2024 Sep 9;34(5-6):517–24. doi: 10.1007/s00572-024-01167-3 (PMC11604758; doi:10.1007/s00572-024-01167-3)
Supplement: Supplementary file 2 — Supplementary Material 2 [file 572_2024_1167_MOESM2_ESM.docx]

Title: Specialized protist communities on mycorrhizal fungal hyphae

Authors: Changfeng Zhang, Stefan Geisen, Roeland L. Berendsen,

and Marcel G. A. van der Heijden

The following supplementary figures are available for this article:

**Fig. S1** Protistan community rarefaction curves.

**Fig. S2** Stereo microspore images of AM hyphae.

**Fig. S3** Differential abundance of protist phyla in the soil and hyphal samples.

**Fig. S4** Relative abundance of Ochrophyta ASVs in soil and hyphal samples.

**Fig. S5** Relative abundance of protist groups in soil and hyphal samples.

**Fig. S6** Overlapped ASVs between soil and hyphal samples.


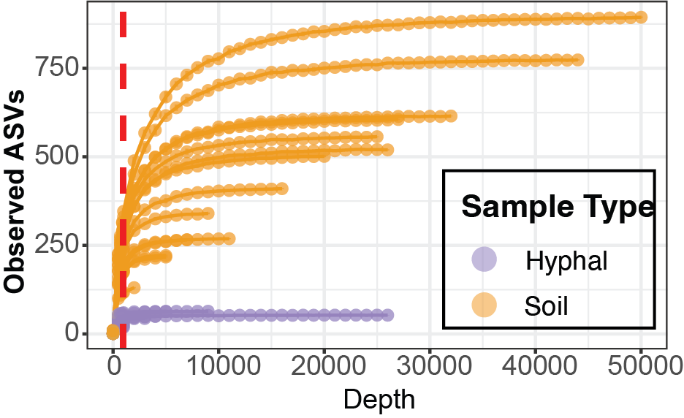


**Fig. S1 Protistan community rarefaction curves.** Colors indicate the types of samples.


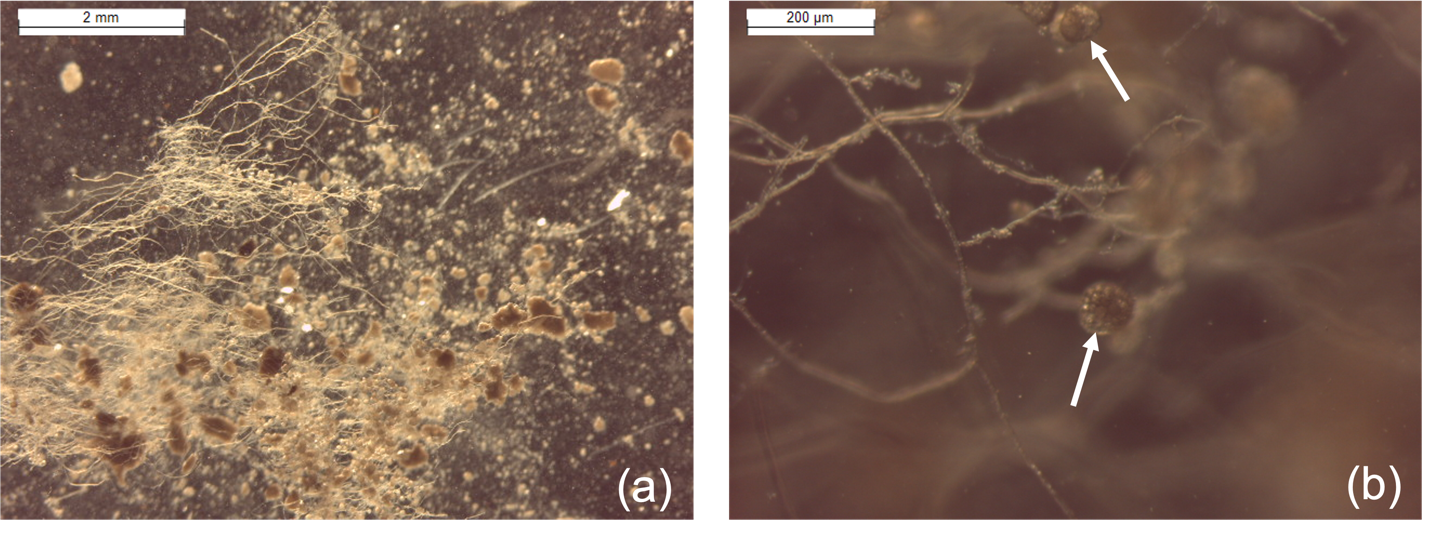


**Fig. S2 Stereo microscope images of AM hyphae.**

**(a)** Images of hyphal samples acquired from COMP5 (Scale bar, 2 mm). **(b)** AM spores attached to fungal hyphae are indicated by arrows (Scale bar, 200 µm). The morphology of the hyphal samples here was compared with the AM hyphae and spores cultured on agar medium with chicory root organs. Further details can be found in Zhang *et al.* 2024.


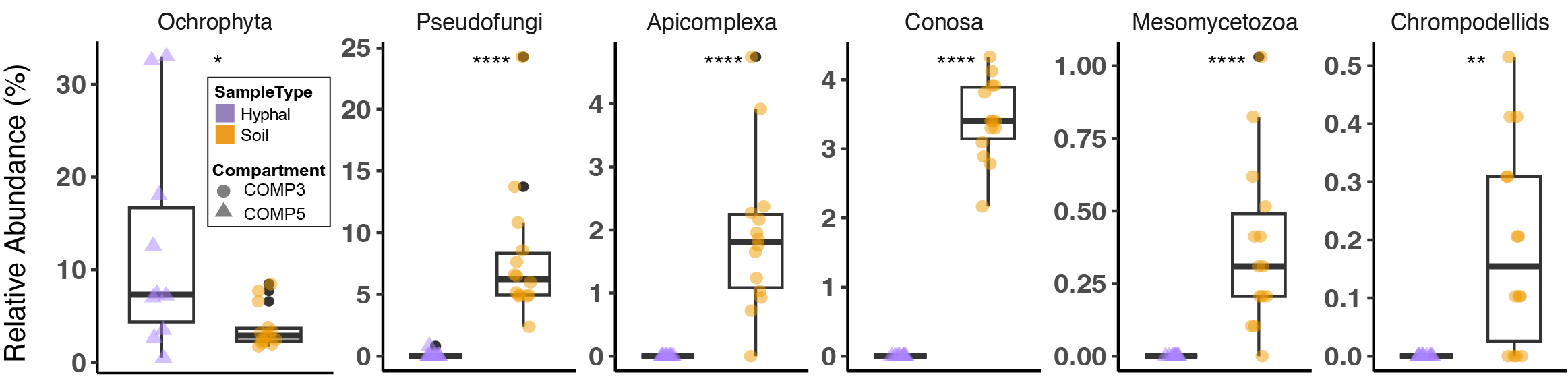


**Fig. S3 Differential abundance of protist phyla in the soil and hyphal samples.**

Only the phyla in either soil or hyphae with more than 1% relative abundance were considered here. The significance levels were determined by Wilcox test (* p < 0.05, ** p < 0.01, *** p < 0.001, **** p < 0.0001). Colors indicate the types of samples. Shapes indicate the compartments from which the samples were ordinated.


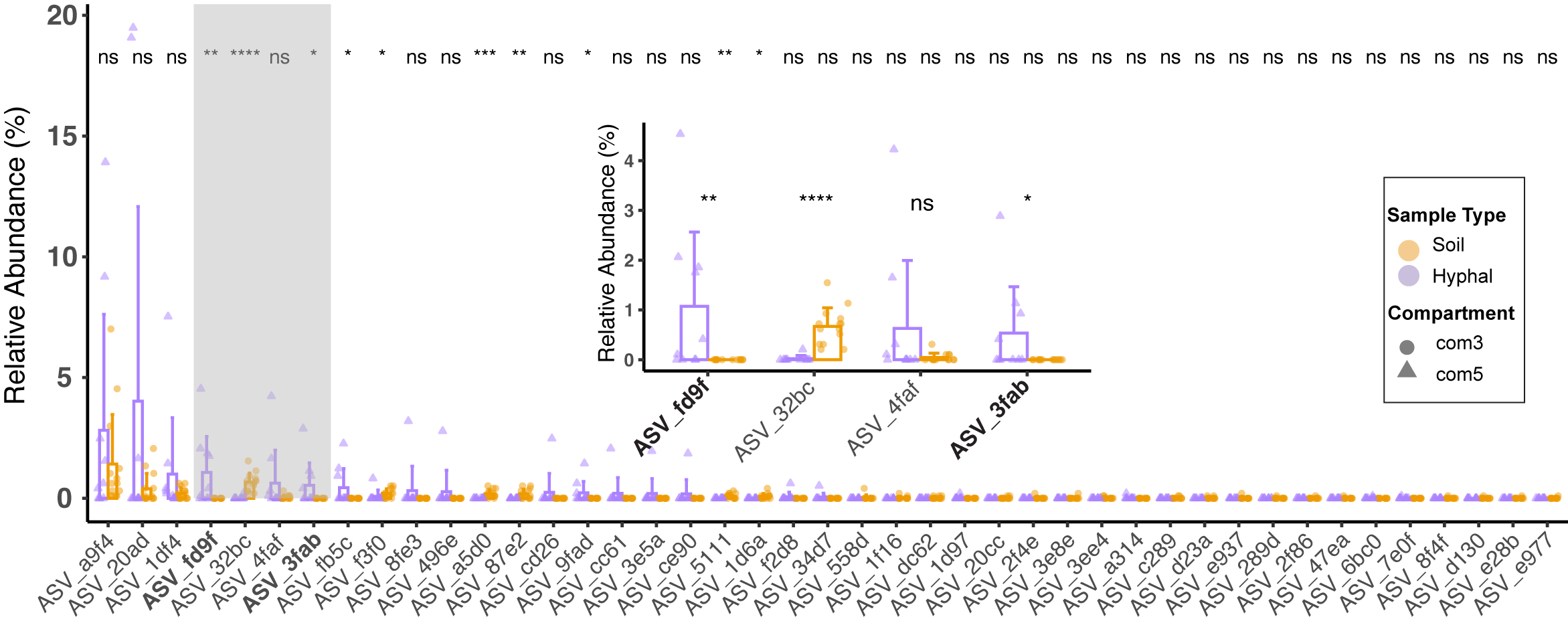


**Fig. S4 Relative abundance of Ochrophyta ASVs in soil and hyphal samples.**

The grey area of the bar plot was zoomed in and are presented within the bar plot. Colors represent the protist groups. Shapes indicate the compartments from which the samples were ordinated. The difference in the relative abundance of soil and hyphal samples was determined by Wilcox-text (* *p* < 0.05, ** *p* < 0.01, *** *p* < 0.001, **** *p* < 0.0001). The hyphae significantly associated ASVs are highlighted with bold font.


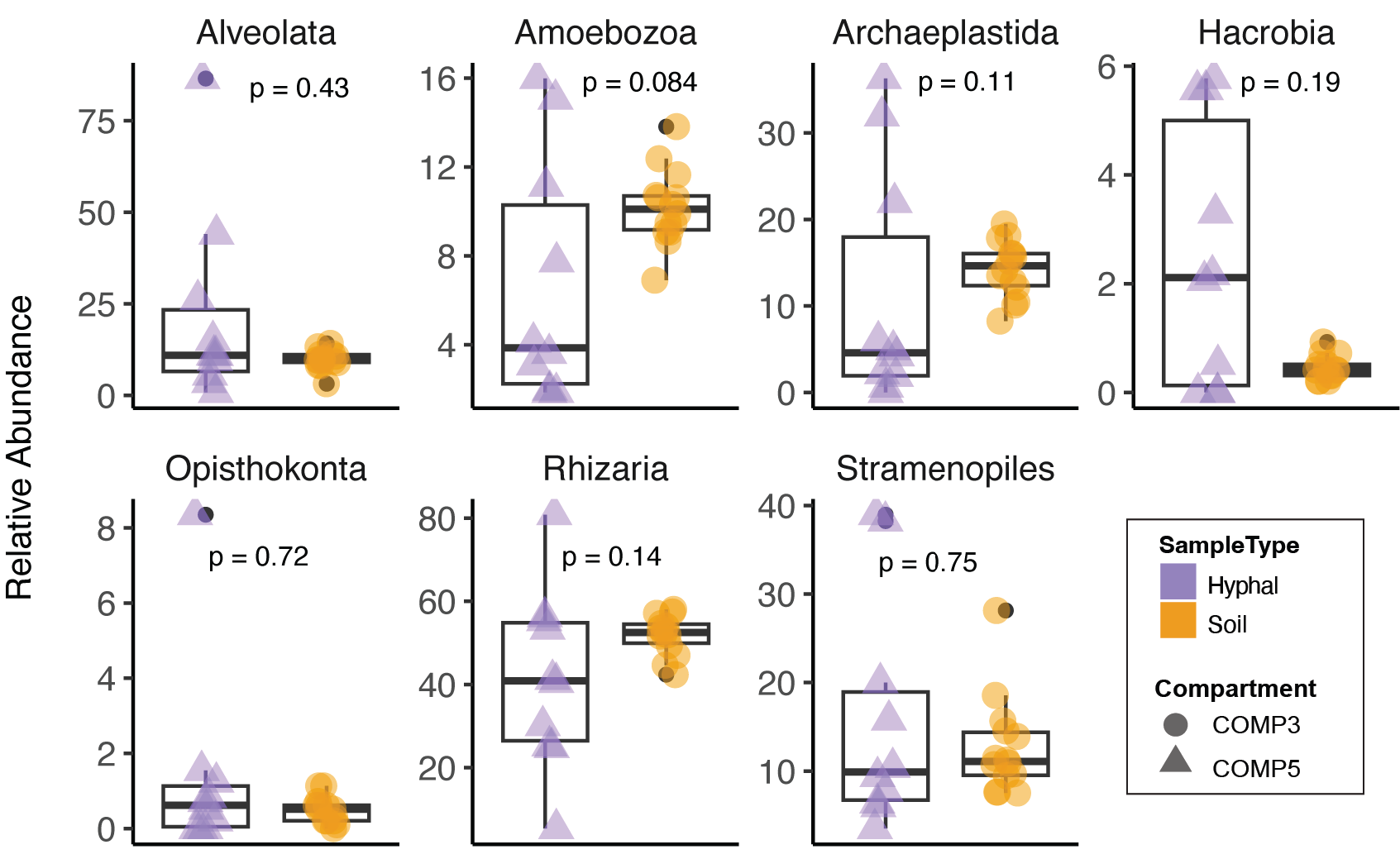


**Fig. S5 Relative abundance of protist groups in soil and hyphal samples.**

The box plots show the difference in relative abundance between soil and hyphal samples, as determined by the Wilcox-text. Colors represent the protist groups. Shapes indicate the compartments from which the samples were ordinated.


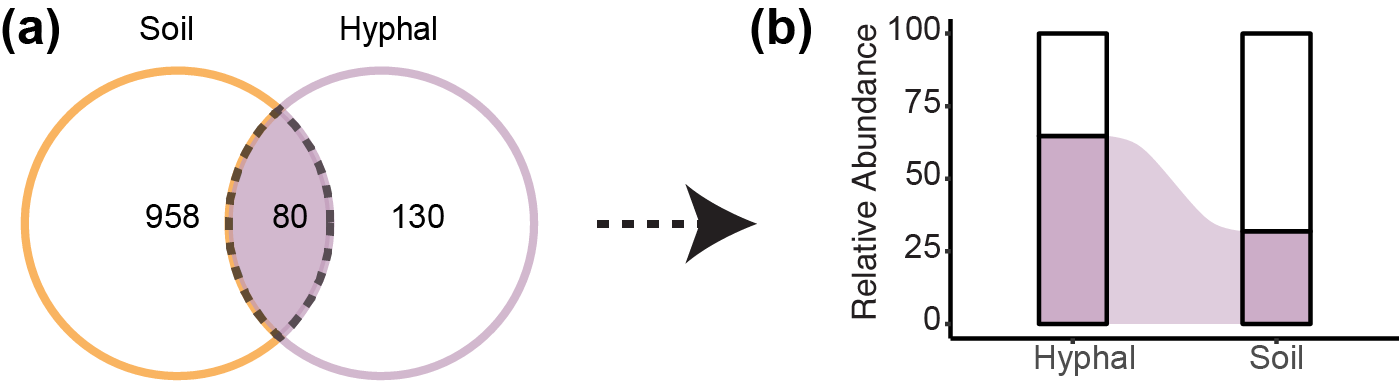


**Fig. S6** Overlapped ASVs between soil and hyphal samples.

**(a)** Venn diagram of hyphal and soil protist ASVs. Numbers represent the amount of ASVs. Colors indicate the sample types. **(b)** Sanky plot of shared ASVs RA in the hyphal and soil samples. The color depicted the relative abundance of shared ASVs in hyphal or soil samples.
